# Intra‑articular tranexamic acid improves 1‑ and 3‑month functional recovery but does not reduce early post‑operative pain after arthroscopic ACL reconstruction: A triple‑blind randomised trial

**DOI:** 10.1002/jeo2.70460

**Published:** 2025-10-09

**Authors:** Mohammad Ayati Firoozabadi, Behzad Nezhadtabrizi, Mohammad Poursalehian, Mohammadreza Razzaghof, Hamed Naghizadeh, Mohammad Soleimani, S. M. Javad Mortazavi

**Affiliations:** ^1^ Joint Reconstruction Research Center Tehran University of Medical Sciences Tehran Iran; ^2^ Department of Orthopedic Surgery Imam Khomeini Hospital Complex Tehran University of Medical Sciences Tehran Iran

**Keywords:** anterior cruciate ligament reconstruction, patient‐reported outcome measures, postoperative pain management, tranexamic acid

## Abstract

**Purpose:**

Our aim is to investigate whether an intra‐articular injection of tranexamic acid (TXA), administered alongside a standard multimodal analgesic cocktail, improves postoperative pain control and functional recovery.

**Methods:**

This was a randomised, triple‐blind, placebo‐controlled clinical trial conducted from April 2022 to October 2023 at a tertiary care center. One hundred patients with isolated, unilateral, chronic ACL tears were randomised 1:1 to receive either an intra‐articular injection containing TXA (1 g), morphine sulphate, ketorolac, lidocaine and normal saline (intervention group) or an identical injection without TXA (control group). All surgeries were performed using hamstring autografts and standardised surgical and postoperative protocols. All patients received 15 mg/kg intravenous TXA in addition to intra‐articular TXA. The primary outcomes were pain scores (Visual Analogue Scale, VAS) at predefined time points up to 3 months. The secondary outcomes were Knee Injury and Osteoarthritis Outcome Score (KOOS), Lysholm scores, range of motion, swelling and morphine consumption.

**Results:**

Postoperative pain scores did not differ significantly between groups at any time point (*p* > 0.05). Both groups demonstrated significant within‐group improvements over time in KOOS subscores (*p* < 0.001) and Lysholm scores (*p* < 0.001). Postoperative morphine consumption was similar in both groups (*p* > 0.05). The TXA group had better Lysholm scores at 1 month (85.8 ± 9.1 vs. 79.6 ± 13.9; *p* < 0.01). The TXA group had significantly better KOOS subscores and Lysholm scores at 3 months with moderate effect size (*p* < 0.01). No serious complications were observed in either group.

**Conclusion:**

Intra‐articular TXA did not significantly reduce early postoperative pain or opioid use when added to a multimodal analgesic regimen and intravenous TXA in arthroscopic ACL reconstruction. However, it was associated with modest improvements in functional recovery at 1 and 3 months postoperatively, as measured by KOOS and Lysholm scores.

**Level of Evidence:**

Level I.

**Abbreviations**
ACL: anterior cruciate ligament
ACLR: anterior cruciate ligament reconstruction
ADL: activities of daily living
ASA: American Society of Anesthesiologists
BMI: body mass index
KOOS: Knee Injury and Osteoarthritis Outcome Score
MCID: minimal clinically important difference
PROMs: patient‐reported outcome measures
QoL: quality of life
RCT: randomised controlled trial
SPSS: Statistical Package for the Social Sciences
TXA: tranexamic acid
VAS: Visual Analogue Scale

## INTRODUCTION

Arthroscopic anterior cruciate ligament (ACL) reconstruction is a frequently performed procedure, critical for restoring knee stability and function in active individuals [8, 25]. Despite advances in surgical techniques and anaesthesia, optimal postoperative pain control remains a priority to facilitate early mobilisation and rehabilitation [3, 4]. To address this, clinicians often employ multimodal pain management strategies, incorporating various analgesics and adjuvants administered intraoperatively or postoperatively [10, 12, 17, 26].

Tranexamic acid (TXA) has been widely studied in orthopedic surgery for its blood‐conserving properties, with additional evidence suggesting potential benefits for decreasing inflammation and postoperative pain [5, 23, 24, 27]. Multiple randomised clinical trials (RCTs) have suggested that TXA can reduce hemarthrosis, swelling, postoperative pain and improve function after ACL reconstruction [1, 18]. The systematic reviews of these RCTs suggested that intravenous TXA is more potent than intra‐articular route for ACLR [1, 18]. A meta‐analysis by Poursalehian et al. found that combining different routes of TXA would increase its efficacy in arthroplasty [24]. Previous studies have evaluated intra‐articular TXA, but they did not evaluate its efficacy on pain levels in combination with other pain‐killer drugs and intravenous TXA [18].

Accordingly, we conducted an RCT to evaluate whether a single‐dose intra‐articular injection of TXA, administered alongside a standard pain cocktail and intravenous TXA, could improve PROMs and pain control following arthroscopic ACL reconstruction. We hypothesised that incorporating TXA would provide clinical improvements in pain control, with the potential to improve postoperative PROMs.

## METHODS

### Trial design

This study was a randomised, triple‐blind, placebo‐controlled clinical trial evaluating the efficacy of intra‐articular TXA in combination with ketorolac, lidocaine, and morphine in patients undergoing arthroscopic ACLR. Participants were randomised in a 1:1 ratio to receive either the combination injection (intervention) or a controlled injection. The trial protocol was registered with the Iranian Registry of Clinical Trials (Registration No.: IRCT20221230056983N1) and conducted at the Imam Khomeini Hospital Complex, a tertiary care center from April 2022 to October 2023.

### Participants and settings

Eligible participants were patients aged between 15 and 50 years with an isolated, unilateral, chronic ACL tear scheduled for arthroscopic ACLR using a hamstring autograft. All patients had an American Society of Anesthesiologists (ASA) physical status of I. Exclusion criteria included multi‐ligament injuries, bilateral ACL tears, bony deformities, meniscal injuries, acute ACL tears (<1 month), individuals with a history of haematologic conditions or ischaemic attacks and patients receiving anticoagulant therapy.

Meniscal injuries were identified through preoperative magnetic resonance imaging (MRI) reviewed by both the operating surgeon and a musculoskeletal radiologist. Patients with definitive meniscal tears (Grade III signal extending to the articular surface) were excluded. However, intrasubstance signal changes not contacting the articular surface (e.g., Grade I–II lesions) were not considered exclusion criteria.

Patients who met eligibility criteria were informed about the study and provided written informed consent. All surgical procedures were performed by the same senior knee surgeon (M.A.F.) and surgical team, under spinal anaesthesia and using a thigh tourniquet (deflated at surgery end). No drains were used postoperatively. Patients stayed in the orthopedic ward overnight for standard postoperative monitoring.

### Interventions

Participants were randomised into two groups:

- Intervention Group: Received an intra‐articular injection containing 1 g of TXA (10 cc), 5 mg of morphine sulphate (0.5 cc), 30 mg of ketorolac (1 cc), 100 mg of lidocaine (5 cc) and 23.5 cc of normal saline at the end of surgery after skin closure (40 cc overall).
- Control Group: Received an intra‐articular injection of 5 mg of morphine sulphate (0.5 cc), 30 mg of ketorolac (1 cc) and 100 mg of lidocaine (5 cc) and 33.5 cc of normal saline at the end of surgery after skin closure (40 cc overall).

All patients underwent diagnostic arthroscopy at the beginning of the procedure. Any patients with unanticipated intraoperative findings, including high‐grade meniscal tears or cartilage lesions (Outerbridge Grade III or IV), were excluded from the study and replaced to maintain the sample size. Cartilage surfaces of the femoral condyles, tibial plateau and patella were systematically evaluated arthroscopically to confirm the absence of significant degenerative changes. All patients underwent arthroscopic ACLR using the trans‐portal anatomical technique via two portals, with femoral and tibial fixation (EndoButton and interference screw, respectively). All patients received 1.5 g intravenous TXA, preoperatively.

To ensure even intra‐articular distribution of the analgesic agents and maintain blinding between groups, normal saline was added to bring the total volume of the injection to 40 mL. This volume was chosen to match volumes used in prior studies and allow for consistent administration without causing capsular overdistension, and also to reduce TXA toxicity for the cartilage [22]. All injections were administered slowly to minimise intra‐articular pressure buildup.

Postoperatively, the knee was wrapped with an elastic bandage, and patients were encouraged to begin knee flexion and extension exercises and mobilise with assistance on the day of surgery. Standardised postoperative medication (e.g., gabapentin, acetaminophen, celecoxib and aspirin prophylaxis) was provided, and patients were discharged the following day. All patients received standardised multimodal analgesia during their hospital stay as per institutional protocol. Morphine consumption within the first 24 h postoperatively was recorded. Oral analgesic use after discharge was not controlled or systematically recorded, and patients were instructed to use over‐the‐counter pain medications as needed.

### Outcomes

The primary outcome was pain assessment. Pain was measured using the Visual Analogue Scale (VAS) at 1 h, 2 h, 3 h, 6 h, 12 h, 1 week, 1 month and 3 months postoperatively. VAS pain scores were assessed by a blinded research assistant. During hospitalisation, pain was assessed at rest. At 1 and 3 months, patients were evaluated during in‐person follow‐up visits and asked to rate their average pain over the past 24 h using a 10‐cm VAS. Pain scores were recorded for standardised activity (walking 10 m). For patients who did not attend in‐person follow‐up, assessments were conducted via telephone using a standardised questionnaire.

Secondary outcomes included a range of clinical and functional measures. The Knee Injury and Osteoarthritis Outcome Score (KOOS) was evaluated preoperatively, 1 month and 3 months postoperatively. Higher KOOS score indicates better function. The Lysholm Knee Scoring Scale was assessed preoperatively and at 1, 4 and 12 weeks. Range of motion was measured using a goniometer both preoperatively and at 12 weeks postoperatively. Swelling was evaluated by measuring the circumference of the knee and malleolar regions at the same time points. Knee circumference was measured at the suprapatellar pouch using a flexible measuring tape with the patient in a supine position and the leg extended and relaxed. The tape was applied snugly but without compressing the soft tissues, and measurements were performed by a single blinded assessor to ensure consistency. Additionally, morphine consumption was recorded for the first 24 h following surgery to quantify postoperative pain management. Complications, including infection, nerve damage and arthrofibrosis, were monitored throughout the study period. All outcome assessments were conducted by a researcher who was blinded to the group assignments, ensuring objective and unbiased data collection.

### Sample size

The sample size was calculated based on detecting a minimal clinically important difference (MCID) of 1.5 mm on the VAS for knee pain after surgery, as reported in previous studies [13]. To achieve a power of 80% and a significance level of 0.05, a total of 90 patients were required. We increased the sample size to account for loss to follow up to 100 patients.

### Randomisation and blinding

Block randomisation with a block size of four was generated via an online randomisation tool. Group allocations were placed in sequentially numbered, sealed, opaque envelopes to ensure concealment. An operating room technician not involved in the study opened the envelope after the patient was prepared for surgery. The injections were prepared by this technician once the surgical team had left the operating room, ensuring that the surgeon was blinded to group assignment. Participants and the outcome assessor were also unaware of treatment allocation, achieving a triple‐blind design.

### Statistical methods

Statistical analysis was performed using SPSS version 26 (IBM Corp., Armonk, NY, USA). Baseline characteristics were compared between groups using the Chi‐square test or Fisher's Exact test for categorical variables. The Kolmogorov–Smirnov test was applied to assess data normality. Parametric data were analysed using independent samples t‐tests, while non‐parametric data were evaluated with the Mann–Whitney *U*‐test. For continuous outcomes, such as KOOS and Lysholm scores, effect sizes with 95% confidence intervals were calculated using Cohen's *d*, where values of 0.2, 0.5 and 0.8 indicated small, medium and large effects, respectively. A *p*‐value ≤ 0.05 was considered statistically significant. Analyses were performed on an intention‐to‐treat basis with no planned interim or subgroup analyses.

### Ethical considerations

The study protocol was approved by the Institutional Review Board [IR.TUMS.IKHC.REC.1401.134]. The trial was conducted in accordance with the Declaration of Helsinki and Good Clinical Practice guidelines. Written informed consent was obtained from all participants before enrolment. No interim analyses were conducted, and no formal stopping guidelines were established, as no adverse events necessitated early termination of the trial.

## RESULTS

### Patient enrolment and follow‐up

**Figure 1:**
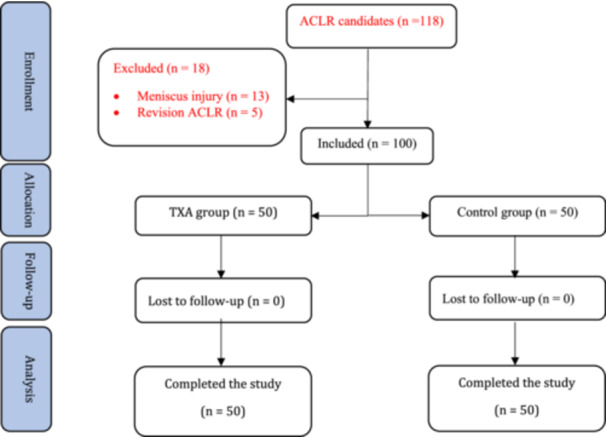
CONSORT flow diagram of patient enrolment, allocation, follow‐up, and analysis. A total of 118 patients were screened for eligibility; 100 were randomised (50 to the tranexamic acid (TXA) group and 50 to the control group), and all completed the trial.

A total of 118 patients were assessed for eligibility, of whom 100 met the inclusion criteria and were randomised into the TXA group (*n* = 50) or the control group (*n* = 50). None of these patients were lost to follow‐up, resulting in a final analysis of all 100 randomised participants (Figure 1).

### Demographics and baseline characteristics

Baseline demographic, clinical and surgical characteristics were comparable between the TXA and control groups, including age, sex, BMI, time since injury and side dominance (Table 1). Preoperative flexion, circumference measurements, and operative parameters (e.g., tourniquet time and pressure) also showed no significant differences between groups. Preoperative assessments—including flexion difference between the affected and unaffected knees, malleolar circumference difference, and knee circumference difference—were also comparable (all *p* > 0.05). Likewise, surgical parameters such as tourniquet time, tourniquet pressure, and operation duration showed no significant differences between groups (all *p* > 0.05, Table 1).

**Table 1:** Patient demographics.

Variable	TXA (*n* = 50)	Control (*n* = 50)
Age (years)	30.2 ± 8.6	30.4 ± 8.2
Sex (female)	4	9
Side (right)	32	28
Weight (kg)	83 ± 12.4	77 ± 14.8
Height (cm)	176.6 ± 8.7	175.1 ± 8.4
BMI (kg/m²)	26.6 ± 4.0	25.0 ± 4.5
Time from ACL injury (months)	6.5 (1–72)	6 (1–72)
Pre‐op flexion difference (°)	0 (0–40)	0 (0–40)
Pre‐op malleolus circumference difference (cm)	0 (–6 to 1)	0 (–2 to 1)
Pre‐op knee circumference difference (cm)	0 (–3 to 2)	0 (–2 to 3)
Tourniquet time (min)	90 (70–130)	90 (70–130)
Tourniquet pressure (mmHg)	270 (240–300)	265 (240–300)
Operation duration (min)	120 (76–160)	120 (70–160)

*Note*: Data are presented as mean ± SD for normally distributed variables and Median (range) for non‐normally distributed variables.

Abbreviations: ACL, anterior cruciate ligament; BMI, body mass index; SD, standard deviation; TXA, tranexamic acid.

### Primary outcomes

**Figure 2:**
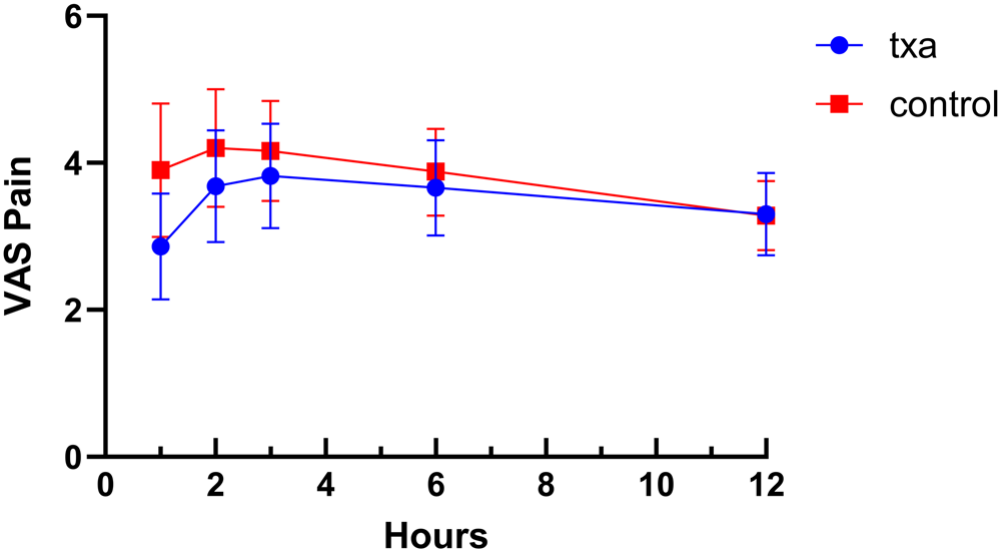
Postoperative VAS pain scores at 1 h, 2 h, 3 h, 6 h and 12 h for the TXA and control groups. Data are presented as mean ± standard deviation. Although the TXA group showed numerically lower pain scores at some early time points, no statistically significant differences were found (*p* > 0.05 for all comparisons). TXA, tranexamic acid; VAS, Visual Analogue Scale.

Pain was measured using the VAS at multiple time points. Although the TXA group showed numerically lower pain scores at 1, 2, 3, and 6 h compared to the control group, these differences did not reach statistical significance (all *p* > 0.05; Figure 2). Pain scores at 1 week, 1 month and 3 months were likewise similar between groups (Table 2).

**Table 2:** Postoperative outcomes.

Outcome	TXA	Control	*p* value
Morphine consumption (mg)	7 (2–9)	7 (1–12)	0.68
Post‐op flexion (°)	127.5 (80–140)	120 (90–145)	0.28
Post‐op malleolus circumference difference (compared to other leg; cm)	10 (0–50)	10 (0–50)	0.69
Post‐op knee circumference difference (compared to other leg; cm)	–1 (–3 to 5)	0 (–3 to 5)	0.88
Post‐op knee circumference difference (compared to pre‐op state; cm)	–1 (–5 to 4)	–1 (–4 to 2)	0.34
VAS pain at 1 week	2.5 (1–5)	2 (0–5)	0.14
VAS pain at 1 month	2 (1–4)	2 (0–4)	0.36
VAS pain at 3 months	1 (0–3)	1 (0–3)	0.72

*Note*: Data are presented as median (range).

Abbreviations: TXA, tranexamic acid; VAS, Visual Analogue Scale.

### Secondary outcomes

Postoperative morphine consumption within the first 24 h did not differ significantly between the TXA and control groups (*p* > 0.05, Table 2).

Knee flexion and the flexion difference between the operated and non‐operated knees were similar at 3 months postoperatively (*p* > 0.05). Swelling, assessed via malleolar circumference and knee circumference differences, showed no statistically significant differences between the groups at 3 months (*p* > 0.05, Table 2).

**Figure 3:**
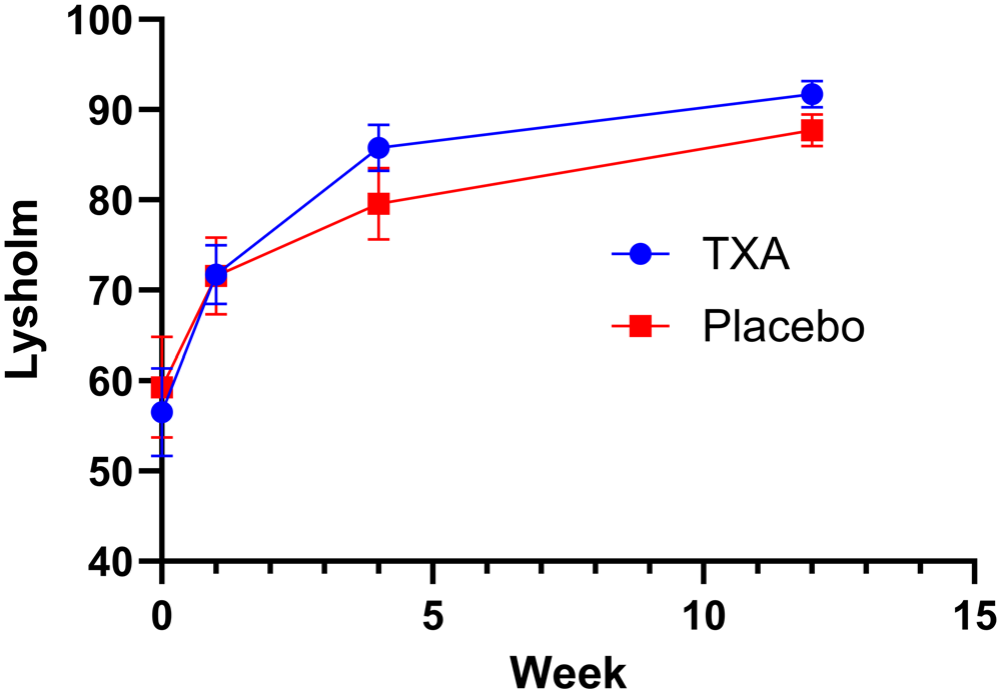
Post‐operative Lysholm score trend in the tranexamic‐acid (TXA) and control groups.

Both groups exhibited a significant increase in Lysholm scores over time (*p* < 0.001 for within‐group changes). At 1 week postoperatively, no significant difference was observed between groups (*p* > 0.05; Figure 3). However, at 1 month, the TXA group had a significantly higher Lysholm score than the control group (moderate effect size, *p* < 0.01). This difference became even more pronounced at 3 months, demonstrating a high effect size favouring the TXA group (*p* < 0.001, Table 3).

**Table 3:** Patient‐reported outcome measures (PROMs).

PROM	Time point	TXA	Control	Effect size (Cohen's *d*, 95% CI)	*p* value
KOOS symptoms	Pre‐op	64.8 (19.8)	68.2 (19.0)	‐	0.39
	1 month	83.4 (12.0)	80.8 (15.4)	0.19 (–0.21 to 0.58)	0.34
	3 months	90.8 (6.8)	87.6 (7.4)	0.46 (0.06–0.86)	**0.024**
KOOS pain	Pre‐op	60.9 (19.1)	62.7 (18.5)	‐	0.62
	1 month	83.9 (11.8)	80.6 (16.2)	0.24 (–0.16 to 0.63)	0.23
	3 months	91.7 (6.3)	88.5 (8.4)	0.43 (0.03–0.82)	**0.036**
KOOS ADL	Pre‐op	68.9 (18.9)	65.5 (19.6)	‐	0.38
	1 month	87.0 (12.2)	80.0 (18.0)	0.45 (0.06–0.85)	**0.026**
	3 months	93.6 (5.4)	88.1 (7.7)	0.83 (0.42–1.24)	**<0.001**
KOOS sports	Pre‐op	49.8 (23.6)	50.0 (19.1)	‐	0.96
	1 month	69.1 (21.6)	75.7 (16.0)	–0.34 (–0.73 to 0.05)	0.08
	3 months	87.1 (7.6)	82.6 (10.6)	0.48 (0.08–0.87)	**0.019**
KOOS QoL	Pre‐op	52.0 (16.2)	55.6 (18.0)	‐	0.30
	1 month	79.4 (14.5)	76.6 (14.7)	0.20 (–0.20 to 0.59)	0.33
	3 months	90.1 (7.0)	85.6 (8.0)	0.60 (0.20–1.00)	**0.003**
Overall KOOS	Pre‐op	59.3 (16.4)	60.4 (16.0)	‐	0.73
	1 month	81.1 (11.6)	78.7 (14.3)	0.18 (–0.21 to 0.57)	0.36
	3 months	90.7 (5.9)	86.5 (7.2)	0.63 (0.23–1.04)	**0.002**
Lysholm	Pre‐op	56.5 (17.2)	59.3 (19.7)	‐	0.44
	1 week	71.7 (11.6)	71.6 (15.1)	0.01 (–0.39 to 0.40)	0.97
	1 month	85.8 (9.1)	79.6 (13.9)	0.54 (0.13–0.93)	**0.009**
	3 months	91.7 (5.2)	87.7 (6.3)	0.77 (0.35–1.19)	**<0.001**

*Note*: Data are presented as mean (SD). Bolded values indicate statistically significant differences in PROMs between study groups.

Abbreviations: ADL, activities of daily living; CI, confidence interval; KOOS, Knee Injury and Osteoarthritis Outcome Score; QoL, quality of life; SD, standard deviation.

Both groups demonstrated significant improvement in all KOOS subscores (Symptoms, Pain, Activities of Daily Living [ADL], Sports, and Quality of Life [QoL]) over time (*p* < 0.001 for within‐group changes). Preoperative KOOS values were similar between the groups (*p* > 0.05). At 1 month, there were no significant between‐group differences in any of the KOOS subscores except for ADL, which was higher in the TXA group (moderate effect size, *p* < 0.05). By 3 months, the TXA group had higher scores across all KOOS subscores than the control group, with moderate‐to‐high effect sizes noted, particularly for KOOS ADL (Table 3).

No complications were observed in either groups including infection, nerve damage and arthrofibrosis.

## DISCUSSION

In this randomised, triple‐blind, placebo‐controlled study, adding intra‐articular TXA to a multimodal analgesic cocktail (morphine, ketorolac, lidocaine and systemic analgesics) and intravenous TXA, did not significantly lower early postoperative pain or morphine use following arthroscopic ACLR. Despite the lack of a statistically significant difference in pain scores up to three months postoperatively, patients receiving TXA exhibited superior functional outcomes at one and three months, as evidenced by higher KOOS subscores and Lysholm scores. It is important to note that these improvements did not reach the MCID thresholds for KOOS and Lysholm subscores, suggesting that the clinical relevance of these functional gains remains uncertain [2, 19].

Several prior RCTs reported reduced pain and improved early knee function with TXA administration in ACLR, yet most differed substantially in TXA dosing strategy, often using intravenous rather than intra‐articular administration, or did not include a comprehensive multimodal pain regimen [6, 7, 9, 11, 14, 16, 21]. For example, Karaaslan et al. [11], Chiang et al. [6], and Ma et al. [16] found that TXA significantly reduced pain; however, the lack of additional intra‐articular agents in those studies makes direct comparisons challenging. Conversely, Fried et al. and Lee et al. observed minimal analgesic benefit from TXA, aligning more closely with our results [9, 14]. One possibility is that our robust multimodal analgesic approach created an analgesic “ceiling effect,” potentially obscuring additional pain control benefits from TXA in the immediate postoperative period.

Regarding PROMs, no previous RCTs specifically examined the KOOS in the setting of ACLR with TXA. However, several investigations have assessed Lysholm scores following ACLR [11, 16, 21]. For instance, Pande and Bhaskarwar demonstrated that intravenous TXA administration was associated with improved Lysholm scores at 2, 6 and 12 weeks postoperatively [21]. Similarly, Karaaslan et al. found significantly higher Lysholm scores at 2 and 4 weeks postoperatively in a TXA group compared to controls, although their methodology and dosing regimen differed from ours [11]. Ma et al. likewise observed better Lysholm scores with TXA usage [16]. These findings align in part with our results, where the addition of TXA facilitated enhanced Lysholm scores at later time points.

The observed functional improvements may be explained by several potential mechanisms. While TXA is primarily known for its antifibrinolytic action, emerging evidence suggests it also possesses anti‐inflammatory properties when used intra‐articularly [15, 20, 28]. TXA may help stabilise the intra‐articular environment by reducing fibrin deposition, limiting hemarthrosis, and subsequently attenuating synovial inflammation and joint irritation [6, 16]. Subclinical inflammation and residual blood products within the joint can impair range of motion, hinder early rehabilitation, and negatively impact patient‐reported outcomes. Therefore, even in the absence of a measurable reduction in acute postoperative pain, the improved joint homoeostasis in the TXA group may have facilitated more effective early mobilisation and rehabilitation, contributing to better subjective functional recovery. However, it should be emphasised that these hypothesised mechanisms were not directly assessed in our study, and further research involving synovial fluid biomarkers, early imaging, and serial functional assessments is needed to confirm these effects.

### Limitations

This study has some limitations that warrant consideration. First, we did not assess inflammatory or biochemical markers that could more directly elucidate the mechanism by which TXA may influence joint recovery. Second, we focused on isolated ACL injuries treated with hamstring autografts; results may not be generalisable to patients with multi‐ligament injuries or different graft choices (e.g., patellar tendon autografts). Third, although we used a triple‐blind design, variations in individual pain thresholds and rehabilitation adherence could influence subjective outcomes like VAS and PROMs. Also, both groups received a standardised and potent multimodal analgesic regimen, including intra‐articular morphine, ketorolac and lidocaine. This likely created a ceiling effect, which may have obscured any additional analgesic benefit from intra‐articular TXA. While ethically necessary for adequate pain control, this design limits our ability to isolate the analgesic effects of TXA alone. Also, we assessed swelling and knee range of motion only at 3 months postoperatively. As such, our study does not capture the early intra‐articular effects of TXA on joint hemarthrosis, which may influence pain, swelling, and mobility during the initial postoperative period. Although we included patient‐reported outcome measures such as KOOS and Lysholm scores at 1 and 3 months, these tools may not accurately reflect functional recovery during the early phase of recovery.

Also, concerns may be raised regarding increased joint effusion from the intra‐articular volume (40 mL); none of the patients in either group experienced postoperative complications such as significant joint swelling, limited range of motion due to effusion or the need for joint aspiration. The injection was delivered under low pressure following capsular closure, and the knee was not distended beyond physiological limits.

Despite these limitations, our findings suggest that while TXA may not substantially reduce early pain in the context of an already potent multimodal analgesic strategy, it could enhance functional recovery over the first few months post‐ACLR. Future studies should explore alternative dosing regimens, including multiple intra‐articular injections or higher doses of TXA, to determine whether these approaches yield greater benefits in pain control or functional outcomes. Investigation into the biochemical mechanisms of TXA's anti‐inflammatory effects, using serum or synovial fluid markers, could also clarify how TXA optimises joint homoeostasis. Furthermore, long‐term follow‐up studies could determine if the observed functional improvements translate into sustained benefits or decreased complication rates over time.

## CONCLUSIONS

In conclusion, intra‐articular administration of TXA, when added to intravenous TXA and a multimodal analgesic regimen, did not significantly reduce early postoperative pain or opioid consumption following arthroscopic ACLR. However, patients who received TXA exhibited modest improvements in functional recovery at 1 and 3 months postoperatively. Given the limitations of the study design, these findings should be interpreted with caution.

## AUTHOR CONTRIBUTIONS

Mohammad Ayati Firoozabadi, Behzad Nezhadtabrizi, and Mohammad Poursalehian wrote the first draft. Mohammadreza Razzaghof and Hamed Naghizadeh did the data gathering. Mohammad Soleimani did the analysis. S. M. Javad Mortazavi supervised the project. All authors participated in revision of this manuscript.

## CONFLICT OF INTEREST STATEMENT

The authors declare no conflicts of interest.

## ETHICS STATEMENT

The study protocol was approved by the Institutional Review Board [IR.TUMS.IKHC.REC.1401.134]. All patients consent to participate in the study.

## Data Availability

Available with a reasonable request from corresponding author's email.
